# Photophysical Study of Polymer-Based Solar Cells with an Organo-Boron Molecule in the Active Layer

**DOI:** 10.3390/ma8074258

**Published:** 2015-07-13

**Authors:** Sergio Romero-Servin, Manuel de Anda Villa, R. Carriles, Gabriel Ramos-Ortíz, José-Luis Maldonado, Mario Rodríguez, M. Güizado-Rodríguez

**Affiliations:** 1 Centro de Investigaciones en Óptica, A.P. 1-948, León, Gto. 37150, Mexico; E-Mails: sromero@cio.mx (S.R.-S.); manueldeandavilla@gmail.com (M.A.V.); jlmr@cio.mx (J.-L.M.); mrodri@cio.mx (M.R.); 2 Centro de Investigación en Ingeniería y Ciencias Aplicadas (CIICAp), Universidad Autónoma del Estado de Morelos, Av. Universidad No. 1001, Col. Chamilpa, Cuernavaca, Mor. C.P. 62209, Mexico; E-Mail: marisolguizado@uaem.mx

**Keywords:** organic photovoltaics, borinate, polythiophenes, transient absorption, bulk heterojunction

## Abstract

Our group previously reported the synthesis of four polythiophene derivatives (P1–P4) used for solar cells. The cells were prepared under room conditions by spin coating, leading to low efficiencies. However, after the addition of 6-nitro-3-(E)-3-(4-dimethylaminophenyl)allylidene)-2,3-dihydrobenzo[d]-[1,3,2] oxazaborole (M1) to their active layers, the efficiencies of the cells showed approximately a two-fold improvement. In this paper, we study this enhancement mechanism by performing ultrafast transient absorption (TA) experiments on the active layer of the different cells. Our samples consisted of thin films of a mixture of PC_61_BM with the polythiophenes derivatives P1–P4. We prepared two versions of each sample, one including the molecule M1 and another without it. The TA data suggests that the efficiency improvement after addition of M1 is due not only to an extended absorption spectrum towards the infrared region causing a larger population of excitons but also to the possible creation of additional channels for transport of excitons and/or electrons to the PC_61_BM interface.

## 1. Introduction

Organic Photovoltaics (OPV) is a very active area of research due to the potential fabrication of these devices at lower cost and using simpler manufacturing techniques, namely spin coating, than their inorganic counterparts. Power conversion efficiencies in OPVs grew up from 0.001% to 1% in the period 1975–1985 [1,2,3] to values in the range 3%–8% commonly achieved nowadays for the bulk heterojunction (BHJ) architecture [4,5,6], although record values on the order of 12% have been reached [7]. Several different approaches are being explored to increase OPVs’ efficiency such as development of new materials, electrodes, architectures, buffer layers and deposition methods [1,8,9].

Among the multiple approaches that can be followed to improve the efficiency of OPVs based in the BHJ architecture, one that has not been commonly explored is the incorporation of organometallic compounds in the active layer [10]. In particular, for photovoltaic applications and other optoelectronics uses, boron bonded to dipolar π-conjugated ligands is able to act as an electron donor, aid in charge transport, or act as hole blocking layer [11,12,13,14,15,16,17,18]. The extended π-conjugation in this family of molecules can push the HOMO level up and simultaneously pull the LUMO level down, causing a reduction in their bandgap [17,19]. This bandgap reduction results in an absorption shift towards the NIR which is attractive for OPVs’ development, since it could result in improved efficiencies due to the broadened absorption.

On the other hand, several poly(p-phenylenevinylene)-, fluorene-, carbazole- and thiophene-based conjugated polymers (among others) have been employed as active layers in OPVs [20,21,22]. To date, polythiophene derivatives, including the regioregular poly(3-hexylthiophene) (P3HT), are among the most commonly employed electron donors in combination with the highly soluble fullerene molecules (PCBM) as electron acceptors for OPVs under the BHJ architecture; their use extends to thin film transistors, light emitting diodes, electrochromic windows and sensors [23]. Polythiophenes offer relatively simple chemical synthesis, the convenience of thin film deposition by spin-coating, and a unique combination of efficient electronic conjugation, and chemical stability [20,21,22,23].

Motivated by the previously discussed properties of polythiophenes and organometallic compounds, we were interested in studying the effects of the incorporation of organometallic molecules into the active layer of different polythiophene based OPVs. Our attention focused on the use of low cost and easily synthesized nonregioregular polythiophenes. For this purpose, in previous work, we synthesized four new polythiophene derivatives (P1–P4) [22,24, (P1 corresponds to PC, P2 to PI, P3 to PD, and P4 to PA)] and built photovoltaic cells with a BHJ architecture containing fullerenes and the organometallic borinate molecule 6-nitro-3-(E)-3-(4-dimethylaminophenyl)allylidene)-2,3-dihydrobenzo[d]-[1,3,2]-oxazaborole (M1) [19]. The series of polymers P1–P4 lack the robustness of other semiconducting materials (*i.e.*, P3HT), widely used in efficient OPVs. This is due mainly to the low regioregularity of P1–P4 [24]; however, since the synthesis of our polythiophenes is based on FeCl_3_ oxidative polymerization, it allows versatile and mild reaction conditions for industrial-scale production at low cost, avoiding the use of expensive catalyzers commonly employed in other synthesis schemes.

**Figure 1:**
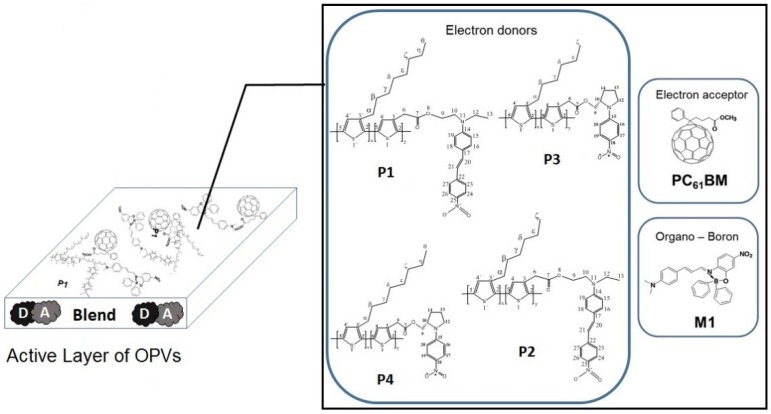
Chemical structures of polythiophene derivatives (P1,P2,P3,P4), PC_61_BM, and M1. D and A correspond to donor and acceptor, respectively.

Figure 1 shows the chemical structure of the materials employed in this study. We found that the low conversion efficiencies of the cells increased by a factor of approximately two upon addition of the borinate [24,25]. At the time of those studies, we did not understand the efficiency enhancement mechanism. In the present work, we study this enhancement by performing ultrafast transient absorption (TA) experiments in thin films of the same composition as those of the active layers of our OPV cells. These ultrafast measurements allow us to compare the photogeneration and dynamics of the excited species, with and without the incorporation of M1. A better understanding of the excited species dynamics after the incorporation of organometallic materials, such as M1, into OPVs can lead to alternative routes for power conversion efficiency enhancements in non-regioregular polymer based cells. Our results suggest that M1 is an absorber of light that not only extends the absorption spectra of the active layer but that could also open new paths for more efficient transport of excitons from the electron-donor polymer to the strong electron acceptor PC_61_BM. Our findings could also be applicable to other polymers of poor charge generation and conducting properties in combination with organic molecules (both easily synthesized and low in cost) to enhance the power conversion efficiency of simple OPVs. The premise is that devices fabricated with low cost materials and very simple methods (in our case a vacuum-free and all-liquid processed OPVs) might certainly comprise low efficiencies (inherent to the limitations of the used materials) but with a minimum of device performance that would be enough for some applications (for instance, in disposable devices) in which very low price is mandatory. Some of the film deposition techniques that can be employed for the fabrication of simple OPVs are spin-coating, roll to roll, doctor blade, drop-casting, inkjet printing, screen printing and spry-coating [26,27,28,29]. In this work, the spin-coating technique was used for the deposition of the active layer while a free-vacuum method based in drop-casting of a metal alloy was used for the cathode deposition. This approach for OPV devices’ fabrication allows an all-liquid process, which so far has not been extensively explored in the literature.

## 2. Results and Discussion

### 2.1. Characterization of Solar Cells Devices 

Copolymers P2 and P3 are derived from 3-hexylthiophene, while P1 and P4 are based on 3-octylthiophene. Copolymers P1 and P2 shared the same monomer 2-(ethyl(4-(4-nitrostyryl)phenyl)amino)ethyl 2-(thiophen-3-yl)acetate, while in copolymers P3 and P4, the monomer is (S)-(−)-1-(4-nitrophenyl)pyrrolidin-2-yl)methyl 2-(thiophen-3-yl)acetate. These monomers are thiophenes functionalized with push-pull chromophores (TF). P1 and P2 have 1.4% and 5% of TF, respectively. The entropy in copolymers P3 and P4 increases because the content of TF (26% and 12%) is higher with respect to P1 and P2. The copolymers P1 and P2 have higher molecular weight and solubility, which in combination with their level of conformational regularity (percentage of dyads in configuration HT and triads in configuration HT-HT) favor a better BHJ morphology. All this leads to higher power conversion efficiency in these OPV’s based on P1 and P2 in comparison to P3 and P4. A summary of the properties of the polymers P1-P4 is presented in Table 1.

**Table 1:** Characteristics of copolymers P1, P2, P3 and P4.

Polymer	Soluble yield (%)	Monomer ratio ^a^	Molecular weight		Configuration					
					Diads (%)			Triads (%)		
			$\bar{Mn}$ (g/mol)	$\bar{Mw}$	HT	HH	HT-HT	TT-HT	HT-HH	TT-HH
P1	79	1.4/98.6	12,600	80,000	76	24	58	14	21	8
P2	63	5/95	24,600	236,000	67	33	43	18	20	19
P3	32	26/74	16,800	106,000	64	36	43	18	19	20
P4	15	12/88	11,000	117,000	68	32	50	15	18	17

Note: ^a^: Ratio between the monomers thiophene functionalized/3-alkylthiophene (alkyl = hexyl or octyl) incorporated into the copolymer.

**Figure 2:**
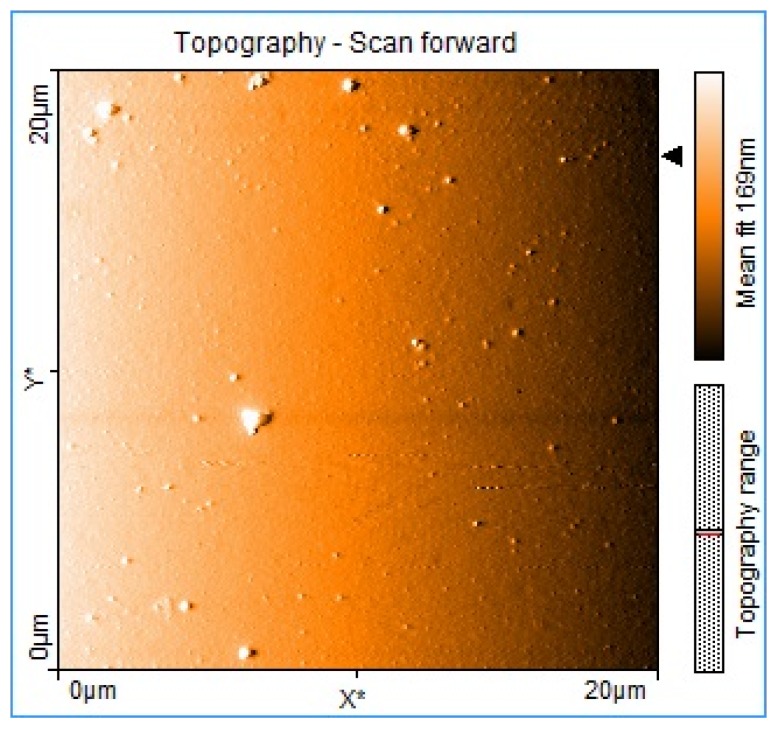
AFM image of active layer of solar cells with the configuration P2:PC_61_BM:M1 (1:2:1 by weight).

The detailed fabrication and characterization of our solar cells based in P1–P4 have been reported elsewhere [24,25]. All our OPV cells were fabricated under room conditions and with an inexpensive method for electrode deposition without the use of a vacuum chamber. Table 2 summarizes the previously obtained results. Although the cells assembled from the polythiophene mixtures show low efficiencies, it is clear that the incorporation of M1 into their active layer resulted in, at least, a two-fold increase in the power conversion efficiency in three of the four cases. The only instance where we did not observe the enhancement effect was for P3. It is worth noting that this particular sample presented a rough surface structure under AFM imaging, in contrast to the other samples. We believe that the low surface quality of P3 could explain the lack of efficiency enhancement after addition of M1. Figure 2 shows a typical AFM image after inclusion of M1 in the film; the surface is smooth with some scattered granules. We also note that the efficiency of the cells based on P4 is approximately five to seven times smaller than those of all the other cells; nevertheless, the enhancement effect of M1 was still present resulting in an improvement factor of two in this particular cell efficiency. As previously mentioned, the physical mechanism that leads to the efficiency enhancement was not well understood before the implementation of the experiments here presented.

**Table 2:** Power conversion efficiency parameters for different organic photovoltaics (OPVs) based on polythiophene derivatives P1–P4 under illumination at 100 mW/cm^2^ [24].

Components (weight ration)	η (%)
P1:PC_61_BM (1:2)	0.17
P1:PC_61_BM:M1 (1:2:1)	0.39
P2:PC_61_BM (1:2)	0.15
P2:PC_61_BM:M1 (1:2:1)	0.41
P3:PC_61_BM (1:2)	0.14
P3:PC_61_BM:M1 (1:2:1)	0.18
P4:PC_61_BM (1:2)	0.02
P4:PC_61_BM:M1 (1:2:1)	0.04

### 2.2. Photophysical Studies of OPVs’ Active Layers

**Figure 3:**
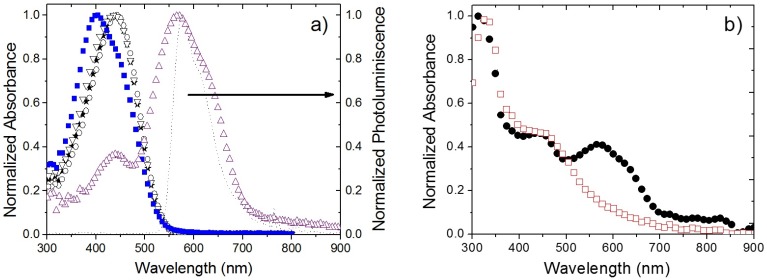
(**a**) Normalized absorption spectra of polythiophene derivative P1 (stars), P2 (inverted triangles), P3 (circles), P4 (squares), and M1 molecule (upright triangles), photoluminescence emission from P1 (dashed line), all spectra were acquired in solution; (**b**) Normalized absorption spectra in solid state of P1:PC_61_BM mixture (1:2 by weight) (open squares) and P1:PC_61_BM:M1 mixture (1:2:1 by weight) (filled circles).

In this section, we report the results, and their interpretation, of the ultrafast transient absorption (TA) measurements performed on our thin films. Some in-depth studies using near infrared femtosecond TA spectroscopy of P3HT films have been published [30,31]. Since our probe beam is generated in an Optical Parametric Amplifier (OPA) system (see Section 3.3), we decided to focus our attention in four particular wavelengths 550 nm, 650 nm, 735 nm, and 900 nm. These wavelengths were selected on the following basis: we expect that the studied polymers will show bleaching at 550 nm because they absorb at this wavelength (see Figure 3a) and this energy is above their respective polaronic bands. The selection of the probe at 735 nm was motivated by the expected presence of a polaronic band for P1–P4 in the range from approximately 700 to 800 nm according to typical values reported for other polythiophenes [32,33]; while the probe wavelength of 650 nm is expected to be between the bleaching and the polaronic TA signals. Finally a probe beam at 900 nm will monitor the Charge Transfer state (CT) formed at the PC_61_BM interface [21,34,35].

The normalized absorption spectra in solution for the polythiophenes (P1–P4) are shown in Figure 3a. P1–P3 (stars, inverted triangles, and circles, respectively) have almost the same spectra and present their maximum absorption at around 440 nm with a FWHM of approximately 120 nm; while P4 (squares) has its maximum at 400 nm and a shoulder at 455 nm. In addition, the same panel includes the absorption spectrum of M1 in solution (upright triangles). On the same Figure 3a, we present the normalized photoluminescence emission curve for P1 in solution (dashed line), for reference; its maximum is located at 570 nm with a FWHM of 80 nm, and it has a shoulder at 625 nm. The emission spectra from P2, P3 and P4 are practically the same than P1 (see Figure S1 in supplementary information). It is observed that there is an overlap between the P1–P4 emission region and the M1 absorption band that, in principle, could lead to an energy transfer mechanism at short interaction range (Förster energy transfer). Nonetheless, this mechanism in our films is rather weak and limited by the fact that the polymers have low fluorescence quantum yields (0.15, 0.13, 0.10, and 0.12 for P1, P2, P3 and P4 in solution, respectively [24]). In addition, it has been reported that for polythiophenes with a low ionization potential, as those considered in this work, photoluminescence quenching is induced when PC_61_BM is added in concentrations above 5% by weight [34,35]. Since our PC_61_BM concentration is much higher, of the order of 200% by weight, we did not expect any noticeable photoluminescence from our samples. We corroborated this assumption since we were unable to measure any light emission from any of the thin film samples.

From Table 2, P1 and P2 have similar power conversion efficiencies, while P3 and P4 have a poorer photovoltaic performance. The higher efficiencies obtained for P1 and P2 are consequence of the larger degree of electronic delocalization that each of these polymers exhibits. As an indirect method to quickly check this property, we measured their third-order optical nonlinearities, *i.e.*, two-photon absorption. It is worth noting that, in polymers, the level of nonlinearities is correlated with such electronic delocalization. By using the TPEF technique [36], we found that the two-photon absorption cross sections at 800 nm (under femtosecond pulse excitation) were 1935, 2579, 945 and 533 GM for P1, P2, P3 and P4, respectively. Thus, the best photovoltaic performance observed in P1 and P2 could be attributed to their larger electronic delocalization in comparison with P3 and P4. Based on the previously mentioned characteristics of the series of polymers P1–P4; we focus our discussion only on the samples based on P1, with and without M1. We corroborated that the results (including transient absorption signals) for the other polythiophenes were similar to those that will be presented for P1. Figure 3b shows the absorption spectra of the polythiophene derivative P1 mixed with PC_61_BM (1:2 by weight, open squares) and of P1:PC_61_BM:M1 (1:2:1 by weight, filled circles), both in solid state. The strong absorption peak seen around 325 nm in both curves is attributed to PC_61_BM [35]. The addition of M1 creates an absorption band starting at approximately 510 nm and extending to 700 nm. This increased absorption is expected to lead to an increment of solar cell power conversion provided that the exciton created in M1 can be dissociated through the interface with PC_61_BM.

**Figure 4:**
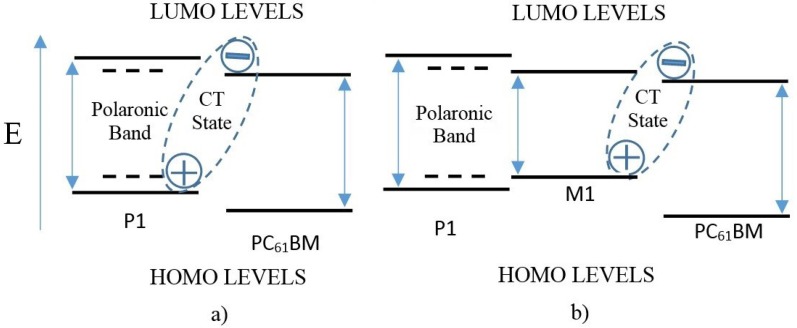
Schematic representation of HOMO and LUMO levels for mixtures: (**a**) P1:PC_61_BM (1:2 by weight) and (**b**) P1:PC_61_BM:M1 (1:2:1 by weight).

When light with energy equal to, or greater than, the optical gap of the donor material is incident on the active layer of an OPV, part of it is absorbed exciting an electron from the HOMO level to above the LUMO level. Due to the Coulombic attraction between the excited electron and the hole left behind, a singlet exciton is created [21,37,38,39]. If the electron is initially excited to a state higher than the LUMO level, it will relax to the LUMO typically in the first couple hundred femtoseconds [21,40]. For the systems under study, the HOMO and LUMO levels are arranged as shown in Figure 4 according to the data presented in Table 3 [24,25]. The energy of the LUMO levels is ordered as E_P1_ > E_M1_ > E_PC61BM_, which favors the direct transport of excitons created in P1 to the interface with PC_61_BM, whether M1 is present or not. Once, at the interface, the electron can be transferred to the PC_61_BM LUMO while the hole remains in the P1 HOMO (or M1 HOMO, when present) creating a CT state [21,34,35,37,38,40,41] that represents the minimum energy required for exciton dissociation. From data in Table 3, we can estimate that the CT state of the mixture containing M1 is approximately 1.52 eV (814 nm), while for the mixture without M1 is 1.85 eV (668 nm). Additionally, when the pump beam excites the sample with M1, there can be photoexcitation of the boronate, creating its own excitons. These species can also be transported to the interface with PC_61_BM, to form a CT state, due to isoenergetic transport because the LUMO levels of M1 and PC_61_BM are partially aligned. Therefore, we expect that the incorporation of M1 to the samples will have at least two significant effects, besides the broadening of the absorption spectrum of the blend: first, it introduces an intermediate state between the LUMO levels of P1 and PC_61_BM that favors exciton or electron transport to the fullerene interface, and second, it reduces the energy gap of the CT state. Thus, the introduction of M1 to the active layer of the BHJ can provide new channels for charge transport to the interface with PC_61_BM. These mechanisms need to be corroborated by time resolved measurements.

**Figure 5:**
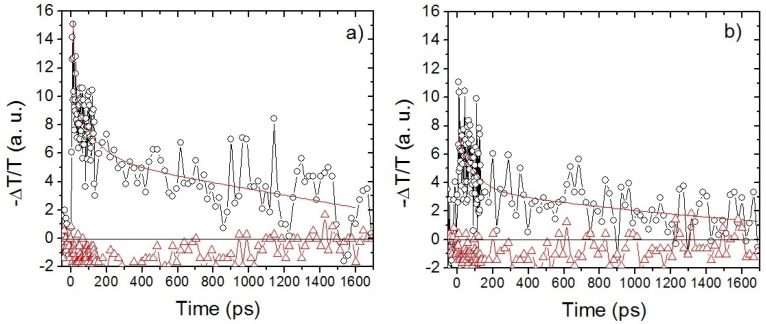
Transient absorption signal of (**a**) P1:PC_61_BM and (**b**) P1:PC_61_BM:M1 with a pump wavelength of 400 nm with energy of 0.7 μJ/pulse and a probe wavelengths of 735 nm (black circles) and 550 nm (red triangles). Red lines are biexponential fittings and the black lines at ΔT/T = 0 are shown as a guide to the eye.

Figure 5 shows transient absorption curves for both mixtures, P1:PC_61_BM in panel (a) and P1:PC_61_BM:M1 in (b), using two different probe wavelengths, 550 nm and 735 nm. Each one of these measurements was carried out independently, and, for different samples with identical preparation, at least eight times; Figure 5 shows the data average. With a 550 nm probe (triangles) both samples undergo ground state bleaching, *i.e.* negative TA signal, and have approximately the same value at all delay times accessible in the experiments. This negative signal is expected at this probe wavelength due to the strong absorption from the ground state of the samples [34,35]. For 735nm (circles), a positive transient signal is observed. We assume that the probe energy falls within the polaronic band of P1 [32,33]. At this probe wavelength, both systems present a sharp increase immediately after excitation, followed by decay within the first nanosecond. Note that the curves do not go down to zero in the time scales that are accessible in our experiment; this means that there is at least one recombination mechanism with a time constant much longer than our experimental temporal range. We have taken these possible mechanisms into account by adding a vertical offset to our fitting curves. The solid curves in Figure 5 are fits to biexponential functions with a constant vertical offset. The estimated decay times for the mixture without M1 are τ_1_ = 12 ps and τ_2_ = 450 ps, while for the sample containing M1 we obtained τ_1_ = 86 ps and τ_2_ = 2260 ps. We observe an increase in the time constants under the presence of M1. We interpret the initial decay (shorter time constant, τ_1_) as arising from the excitons and/or electrons that transfer from either the LUMO level or the polaronic band of P1 to the LUMO level of PC_61_BM (or M1 when it is present); while, for longer times (hundreds of picoseconds and few nanoseconds), the time constant (τ_2_) corresponds to a relaxation of the remaining excitons in the polymer, internal recombination and/or intersystem crossing. We attribute the increase in the time constants (τ_1_, τ_2_) upon incorporation of M1, to the close alignment of the LUMO levels of M1 and PC_61_BM leading to isoenergetic transport; this process generally requires a longer time (hundreds of picoseconds to few nanoseconds) than downhill transport [40], which is the dominant transport mechanism when M1 is absent.

**Table 3:** Electrochemical HOMO and LUMO levels and GAP of P1, M1 and PC_61_BM.

Electrochemical	P1	M1	PC_61_BM
LUMO (eV)	−3.54	−3.66	−3.7
HOMO (eV)	−5.55	−5.22	−6.1
GAP (eV)	2.01	1.56	2.4

Table 4 summarizes the fitted time constants for the TA signals at the different probe wavelengths. In all cases the fit was to a biexponential with a vertical offset. When the probe pulse is set to 650 nm (at the tail of the absorption spectra of M1) we are close to the polaronic band edge of P1 (corresponding to the range 700 and 800 nm [33]). When the probe beam is tuned to 735 nm we expect to be probing the P1 polaronic band, as it was mentioned above. Finally, for the case of a 900 nm probe we obtain a very fast decay (τ_1_) in both samples indicating an efficient transport and dissociation of exciton to the interface with PC_61_BM.

**Table 4:** Time decay constants for transient absorption (TA) signal for the different studied samples.

Probe Wavelength (nm)	Time Constants of biexpontial decay			
	P1:PC_61_BM		P1:PC_61_BM:M1	
	Short τ_1_ (ps)	Long τ_2_ (ns)	Short τ_1_ (ps)	Long τ_2_ (ns)
650	17	0.36	47	2.31
735	12	0.45	86	2.26
900	4	1.25	0.92	0.212

We conclude that the presence of M1 in this system helps to more efficiently take the photoinduced excitons or electrons in the polythiophene to the PC_61_BM interface due to the LUMO alignment between the two compounds. In addition, M1 contributes to the cell efficiency by producing its own excitons that, given the LUMO alignment for the system, are transported to the interface with PC_61_BM. In order to establish a connection with a system widely used in OPVs, we also prepared two more samples with a mixture of PC_61_BM and MEH-PPV, again one with and the other without M1. MEH-PPV is a widely used polymer in organic optoelectronics that provides a well characterized material for comparison. Due to experimental restrictions our MEH-PPV based cells exhibited a rather poor efficiency as compared with state-of-the-art devices; our measured efficiencies were 0.11% for the sample without M1, and 0.36% when M1 is incorporated. Although the MEH-PPV cells had such low efficiencies, a three-fold enhancement of this parameter was measured upon addition of M1 [25]. This demonstrates that the underlying enhancement mechanism is not restricted to our specific polythiophenes. While we do not expect to have such large enhancements, or perhaps not to have any, for state-of-the-art devices which comprise the use of donor materials of optimized electronic characteristics, we wanted to understand the enhancement mechanism and to explore the viability of organometallic molecules in OPV devices. Although some of the obtained numerical values varied, in general, we observed similar behaviors for the P1 and MEH-PPV samples upon addition of M1 in the different spectral regions covered by our experiments: energies above, within, and below the expected polaronic band (see supplementary material).

As mentioned before, the efficiencies achieved in our solar cells are relatively poor; this is expected due to the low regioregularity of the synthesized polythiophenes. Nevertheless, even with the low efficiencies obtained, this study gives us a better understanding of the mechanisms behind the observed enhancements. In some lateral preliminary work, we have recently incorporated the molecule M1 into other new materials that are more efficient than those studied here, and have found that it still leads to an efficiency improvement. Finally, as a remark, there are compounds that show much better efficiencies than the ones reported and explained here, like P3HT. We tried to compare our work with this standard but we found that P3HT and M1 could not form films of good optical quality.

In short, for all the samples under study the presence of M1 not only enhanced the absorption range of the BHJ but also created new paths for charge transport from the electron donor polymer to the interface with PC_61_BM due to the positioning of the LUMO levels of M1 with respect to the polaronic band. In parallel, the direct excitation of M1 also contributed to excite species that are transferred efficiently to the interface with PC_61_BM.

## 3. Experimental Section

### 3.1. Polythiophene Derivatives

Figure 1 shows the chemical structure of the different compounds used in this study. The four polythiophene derivatives are: 2-(ethyl(4-(4-nitrostyryl)phenyl)amino)ethyl 2-(thiophen-3-yl)acetate with 3-octylthiophene, poly(3-OT-*co*-3-TPhNO_2_) (P1); 2-(ethyl(4-(4-nitrostyryl)phenyl)amino)ethyl 2-(thiophen-3-yl)acetate with 3-hexylthiophene, poly(3-HT-*co*-3-TPhNO_2_) (P2); 3-hexylthiophene and (S)-(−)-1-(4-nitrophenyl)pyrrolidin-2-yl)methyl 2-(thiophen-3-yl)acetate, poly(3-HT-*co*-3TPyNO_2_) (P3); and 3-octylthiophene and (S)-(−)-1-(4-nitrophenyl)pyrrolidin-2-yl)methyl 2-(thiophen-3-yl)acetate, poly(3-OT-*co*-3-TPyNO_2_) (P4). Details of the synthesis and characterization of these materials are in [24]. This figure also presents the chemical structure of PC_61_BM, and M1 [19]. All commercial chemicals were purchased from Aldrich and Fermont and they were used without purification.

### 3.2. Sample Preparation

Our samples for transient absorption measurements were fabricated to replicate the active layer of the OPV devices reported in references [24,25]. We prepared ten different solutions: one for each of the four polythiophene derivatives (P1–P4) with PC_61_BM in a weight ratio of 1:2, one for each of the four polythiophene derivatives with PC_61_BM and M1 in a weight ratio of 1:2:1, one for MEH-PPV with PC_61_BM in a weight ratio of 1:2, and one of MEH-PPV with PC_61_BM and M1 in a weight ratio of 1:2:1 (see supplementary material). Chloroform was used as solvent for the polythiophene derivatives except for P3 for which toluene was employed because it does not dissolve in chloroform. For the MEH-PPV samples, dichloromethane was used as solvent. Samples were deposited by spin-coating into thin films (between 80 and 120 nm in thickness) on top of glass coated ITO substrates under standard room conditions.

### 3.3. Steady-State and Transient Absorption Experiments

**Figure 6:**
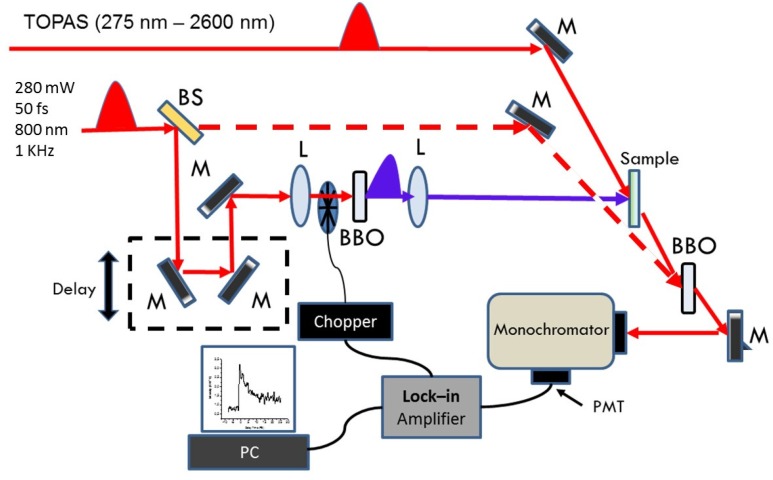
Experimental setup for ultrafast two color transient absorption spectrometer. M, L, BS, and PMT denote mirror, lens, beam splitter, and photomultiplier tube, respectively.

Steady-state absorbance spectra were obtained for all the samples, both in solution and in solid state, using a spectrophotometer (Perkin Elmer, Lambda 900, Waltham, MA, USA) over a range from 300 to 1200 nm. To study the photodynamics of the samples, we implemented a femtosecond transient absorption (TA) two color spectrometer in a pump-probe configuration. In short, we measured the transmitted intensity of a probe pulse through the thin films as a function of its time of arrival to the sample after a more intense pump pulse. The wavelength of the probe pulse could be changed, while the pump pulse was fixed at 400 nm. We define our TA signal as: TA = −ΔT/T_0_ = −(T−T_0_)/T_0_; where T_0_ denotes the transmission through the sample in the absence of the pump pulse, and T is the transmission when the pump pulse is present. According to this definition a positive transient absorption signal is due to an increased absorption of the probe beam in the sample after excitation by the pump pulse, while a negative TA signal indicates photobleaching and/or stimulated emission [34]. Our experimental setup is shown in Figure 6. The laser source consisted of a “Libra” Ti: Sapphire Regenerative Amplifier (800 nm wavelength, 50 fs FWHM pulse width, energy of 3.5 mJ/pulse, 1 kHz repetition rate, Coherent Inc., Santa Clara, CA, USA). The 800 nm beam from the Libra system was split into three parts. The first part, approximately 1 W, was directed into an optical parametric amplifier (TOPAS, Light Conversion, Vilnius, Lithuania) tunable from 285 to 2600 nm; the beam from the TOPAS system was used as the probe pulse. The second part of the fundamental beam, approximately 140 mW, was frequency doubled by a BBO crystal (500 μm thick, 28.1°, from Inrad, Northvale, NJ, USA); the resulting 400 nm light was used as the pump pulse with energy of approximately 0.7 μJ/pulse at the sample. We corroborated that the TA decay traces were not affected by this pump energy level, thus discarding the possibility of nonlinear interactions or artifacts. The transmission of the probe pulse through the sample was measured by a PMT (Hamamatsu RT400U-02, Hamamatsu, Japan) mounted at the exit port of a monochromator (Acton Research, SpectraPro-2500, Trenton, NJ, USA), in combination with a lock-in amplifier. The sensitivity of our PMT cuts at approximately 820 nm. In order to detect probe pulses beyond this wavelength, we implemented a sum frequency generation detection scheme. As shown in Figure 6, the third part of the original beam (dashed red line) from the Libra system was mixed with the probe pulse in a second BBO crystal (200 μm, 29.1° from EKSMA, Vilnius, Lithuania); thus allowing us to convert the IR probe pulse into a spectral range that can be detected by our PMT. A delay line based in a translation stage was used to control the time of arrival of the probe pulse with respect to the pump pulse.

## 4. Conclusions

In this paper, we analyze transient and stationary absorption measurements to elucidate why the incorporation of the organo boron molecule M1 into the active layer of solar cells, based on polythiophene derivatives P1–P4, induces an improvement of the power conversion efficiency by a factor of almost two. We conclude that the incorporation of M1, not only broadens the absorption spectra towards the infrared region but also modifies the temporal constants involved in the observed biexponential decays. For the P1 samples probed at 735 nm, the increase in the decay constants is of up to seven times. We attribute these results to M1 providing an additional path for exciton and/or electron transport to the PC_61_BM interface. We found that excitons can migrate from the excitonic band to the LUMO level of M1, and then to the interface with PC_61_BM, or directly from the LUMO levels of P1 to M1 and then to PC_61_BM. Similar results and trends were obtained for the MEH-PPV system.

## Supplementary Materials

Supplementary materials can be accessed at: http://www.mdpi.com/1996-1944/8/7/4258/s1.

## References

[1] Hoppe H., Serdar Sariciftci N. (2004). Organic solar cells: An overview. J. Mater. Res..

[2] Tang C.W., Albrecht A.C. (1975). Photovoltaic effects of metal-chlorophyll-a-metal sandwich cells. J. Chem. Phys..

[3] Tang C.W. (1986). Two-layer organic photovoltaic cell. Appl. Phys. Lett..

[4] Ohori Y., Hoashi T., Yanagi Y., Okukawa T., Fujii S., Kataura H., Nishioka Y. (2014). Organic solar cells based on ternary blend active layer of two donors PTB7, P3HT and acceptor PC_61_BM. J. Photopolym. Sci. Technol..

[5] Kim J.Y., Kim S.H., Lee H.-H., Lee K., Ma W., Gong X., Heeger A.J. (2006). New architecture for high efficiency polymer photovoltaic cells using solution-based titanium oxide as an optical spacer. Adv. Mater..

[6] Park S.H., Roy A., Beaupré S., Cho S., Coates N., Moon J.S., Moses D., Leclerc M., Lee K., Heeger A.J. (2009). Bulk heterojunction solar cell with internal quantum efficiency approaching 100%. Nat. Photonics.

[7] Li G., Zhu R., Yang Y. (2012). Polymer solar cells. Nat. Photonics.

[8] Sun Y., Welch G.C., Leong W.L., Takacs C.J., Bazan G.C., Heeger A.J. (2012). Solution-processed small-molecule solar cells with 6.7% efficiency. Nat. Mater..

[9] Zhou H., Yang L., You W. (2012). Rational design of high performance conjugated polymers for organic solar cells. Macromolecules.

[10] Wong W. (2009). Challenges in organometallic research–great opportunity for solar cells and oleds. J. Organomet. Chem..

[11] Qiao F., Liu A., Zhou Y., Xiao Y., Yang P.O. (2009). Bulk heterojunction organic solar cell based on a novel fluorescent fluorine-boron complex. J. Mater. Sci..

[12] Pandey R., Zou Y., Holmes R.J. (2012). Efficient bulk heterojunction organic photo-voltaic cells based on boron subphathalocyanine chloride-C70. Appl. Phys. Lett..

[13] Jakle F. (2010). Advances in the synthesis of organoborone polymers for optical, electrical and sensory applications. Chem. Rev..

[14] Qin Y., Kiburu I., Shah S., Jakle F. (2006). Luminescence tuning of organoboron quinolates through substituent variation at the 5-position of the quinolato moiety. Org. Lett..

[15] Cataldo S., Fabiano S., Ferrante F., Preveti F., Patane S., Pignataro B. (2010). Organoboron polymers for photovoltaic bulk heterojunctions. Macromol. Rapid Commun..

[16] Wu Q., Esteghamatian M., Hu N., Popovic Z., Enright G., Tao Y., D’lorio M., Wang S. (2000). Synthesis, structure, and electroluminescence of BR2q(R = Et, Ph, 2-Naphthyland q = 8-Hydroxyquinolto). Chem. Mater..

[17] Li D., Wang K., Huang S., Qu S., Liu X., Zhu Q., Zhang H., Wang Y. (2011). Brightly fluorescent red organic solids bearing boron-bridged π-conjugated skeletons. J. Mater. Chem..

[18] Jakle F. (2006). Lewis acidic organoboron Polymers. Coord. Chem. Rev..

[19] Rodriguez M., Maldonado J.L., Ramos-Ortiz G.J., Lamere F., Lacroix P.G., Farfan N., Ochoa M.E., Santillan R., Meneses-Nava M.A., Barbosa-Garcia O. (2009). Syntehsis and non-linear optical characterization of novel borinate derivatives of cinnamaldehyde. New J. Chem..

[20] Clarke T., Durrant J. (2010). Charge photogeneration in organic solar cells. Chem. Rev..

[21] Cheng Y., Yang C., Hsu C. (2009). Synthesis of conjugated polymers for organic solar cells applications. Chem. Rev..

[22] Chasteen S.V., Sholin V., Carter S.A., Rumbles G. (2008). Towards optimization of device performance in conjugated polymer photovoltaics: Charge generation, transfer and transport in poly(p-phenylene-vinylene) polymer heterojunctions. Sol. Energy Mater. Sol. Cells.

[23] Agina E.V., Ponomarenko S.A., Muzafrov A.M. (2010). Macromolecular systems with the p-type conductivity. Russ. Chem. Bull..

[24] Del-Oso J.A., Maldonado J.L., Ramos-Ortiz G., Rodriguez M., Guizado-Rodriguez M., Escalante J., Frontana-Uribe B.A., Perez-Gutierrez E., Santillan R. (2014). New polythiopene derivatives and enhanced photovoltaic effect by a boron compound blended with them in OPVs cells. Synth. Met..

[25] Salinas J.F., Maldonado J.L., Ramos-Ortíz G., Rodríguez M., Meneses-Nava M.A., Barbosa-García O., Santillan R., Farfán N. (2011). On the use of woods metal for fabricating and testing polymeric organic solar cells: An easy and fast method. Sol. Energy Mater. Sol. Cells.

[26] Yu J., Zheng Y., Huang J. (2014). Towards high performance organic photovoltaic cells: A review of recent development in organic photovoltaics. Polymers.

[27] Kaur N., Singh M., Pathak D., Wagner T., Nunzi J.M. (2014). Organic materials for photovoltaic applications: Review and mechanism. Synth. Met..

[28] Yan J., Saunders B. (2014). Third-generation solar cells: A review and comparison of polymer: Fullerene, hybrid polymer and perovskite solar cells. RSC Adv..

[29] Huang Y., Kramer E.J., Heeger A.J., Bazan G.C. (2014). Bulk heterojunction solar cells: Morphology and performance relathionships. Chem. Rev..

[30] Kandada R.S., Grancini G., Perozza A., Perissinotto S., Fazzi D., Raavi S.S.K., Lanzani G. (2013). Ultrafast energy transfer in ultrathin organic donor/acceptor blend. Sci. Rep..

[31] Guo J., Ohkita H., Benten H., Ito S. (2009). Near-IR femtosecond transient absorption spectroscopy of ultrafast polaron and triplet exciton formation in polythiophene films with different regioregularities. J. Am. Chem Soc..

[32] Ohkita H., Cook S., Astuti Y., Duffy W., Tierney S., Zhang W., Heeney M., McCulloch I., Nelson J., Bradley D.D.C. (2008). Charge carrier formation in polythiophene/fullerene blend films studied by transient absorption spectroscopy. J. Am. Chem. Soc..

[33] Roncali J. (1992). Conjugated poly(thiophenes): Synthesis, functionalization and applications. Chem. Rev..

[34] Piris J., Dykstra T.E., Bakulin A.A., van Loosdrecht P.H.M., Knulst W., Trinh M.T., Schins J.M., Siebbeles L.D.A. (2009). Photogeneration and ultrafast dynamics of excitons and charges in P3HT/PCBM blends. J. Phys. Chem. C..

[35] Cook S., Katoh R., Furube A. (2009). Ultrafast studies of charge generation in PCBM:P3HT blend films following excitation of the fullerene PCBM. J. Phys. Chem. C.

[36] Albota M.A., Xu C., Webb W.W. (1998). Two-photon fluorescence excitation cross sections of biomolecular probes from 690 to 960 nm. Appl. Opt..

[37] Martini B., Smith A.D., Schwartz B.J. (2004). Exciton-exciton annihilation and the production of interchain species in conjugated polymer films: Comparing the ultrafast stimulated emission and photoluminescence dynamics of MEH-PPV. Phys. Rev. B.

[38] Hodgkiss M., Albert-Seifried S., Rao A., Barker A.J., Campbell A.R., Marsh R.A., Friend R.H. (2012). Exciton-charge annihilation in organic semiconductor films. Adv. Funct. Mater..

[39] Benson-Smith J., Goris L., Vandewal K., Haenen K., Manca J.V., Vanderzande D., Bradley D.D.C., Nelson J. (2007). Formation of a ground-state charge-tranfer complex in polyfluorene/[6,6]-phenyl-C_61_ Butyric Acid Methyl Ester (PCBM) blend films and its role in the function of polymer/PCBM solar cells. Adv. Funct. Mater..

[40] Scheblykin G., Yartsev A., Pullerits T., Gulbinas V., Sundstrom V. (2007). Excited state and charge photogeneration dynamics in conjugated polymers. J. Phys. Chem. B.

[41] Vandewal A., Gadisa W.D., Oosterbaan S., Bertho F., Banishoeib I.V., Severen L., Lutsen T., Cleij J., Vanderzande D., Manca J.V. (2008). The relation between open-circuit voltage and the onset of photocurrent generation by charge-transfer absorption in polymer: Fullerene bulk heterojunction solar cells. Adv. Funct. Mater..

