# Supplementary material for: Photophysical Study of Polymer-Based Solar Cells with an Organo-Boron Molecule in the Active Layer
Source: Materials (Basel). 2015 Jul 13;8(7):4258–72. doi: 10.3390/ma8074258 (PMC5455641; doi:10.3390/ma8074258)
Supplement: Supplementary file 1 [file materials-08-04258-s001.pdf]

Supplementary Materials

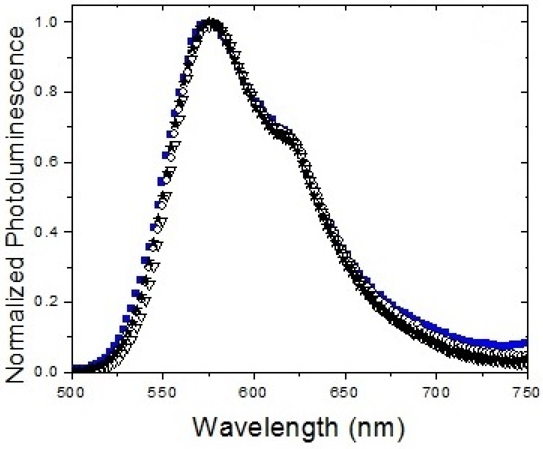

Figure S1. Normalized photoluminescence of polythiophenes P1 (stars); P2 (inverted triangles); P3 (circles); P4 (squares).

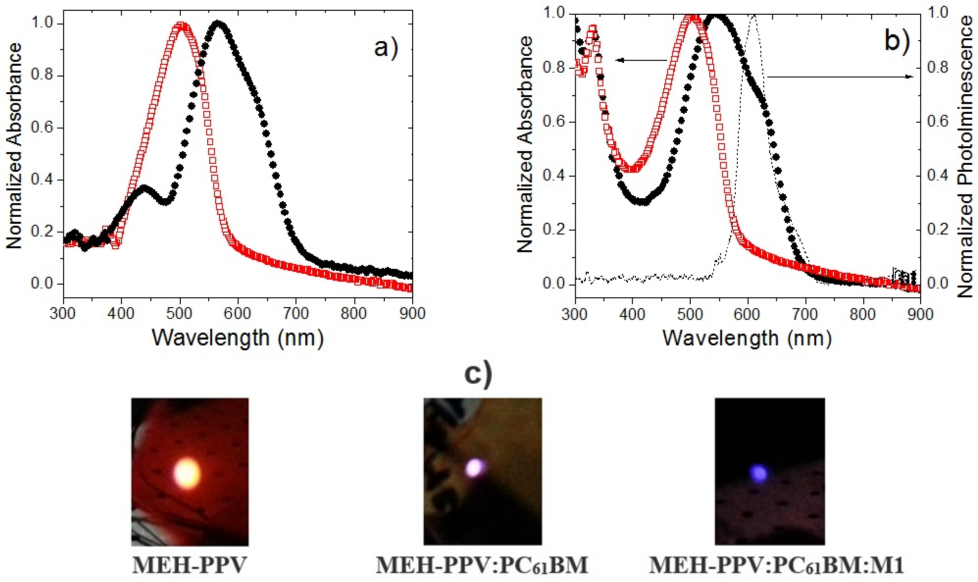

Figure S2. Normalized absorption spectra. (**a**) MEH-PPV (open squares) and M1 (circles), both taken in solution; (**b**) MEH-PPV:$PC_{61}BM$ (1:2 by weight, open squares), MEH-PPV:$PC_{61}BM$:M1 (1:2:1 by weight, filled circles) and normalized photoluminescence emission spectrum of MEH-PPV (dashed line), all the three samples were in solid state; (**c**) Quenched photoluminescence in MEH-PPV by the presence of $PC_{61}BM$ and M1 under 400nm laser illumination.

Addition of M1 to the MEH-PPV:PC61BM mixture broadens the absorption towards the infrared.

The measured TA signal for the MEH-PPV:$PC_{61}BM$ and MEH-PPV:$PC_{61}BM$:M1 are shown in Figure S3. For a 550 nm probe, the signal is negative due to ground state bleaching. At probe wavelength of 650 nm we expect the TA signal to monitor the polaronic band of MEH-PPV, since it extends from 600 to 800 nm. The solid lines shown in Figure S3 are fits to a biexponential function with a vertical offset, Table S1 presents the obtained values for the fitted decay constants.

As with the polythiophene system, the value of the decay constants increases upon incorporation of M1. For the sample without M1 (Figure S3a), the faster time constant (τ_1 = 20 ps) is within the

value of typical of times for Stimulated Emission (SE) in MEH-PPV, followed by a relaxation that extends to around 400 ps, that is in agreement with the longer decay constant, τ_2, measured in our experiments. We propose that the introduction of M1 quenches the SE (see Figure S2c) because the excitons will migrate to the M1 LUMO band, leading to longer TA decay time constants (see Table S2). Once the electrons are in the LUMO of M1, they can be transported to the interface with $PC_{61}BM$; thus M1 is helping to transport excitons to the fullerene interface that otherwise would recombine or transfer to triplet states.

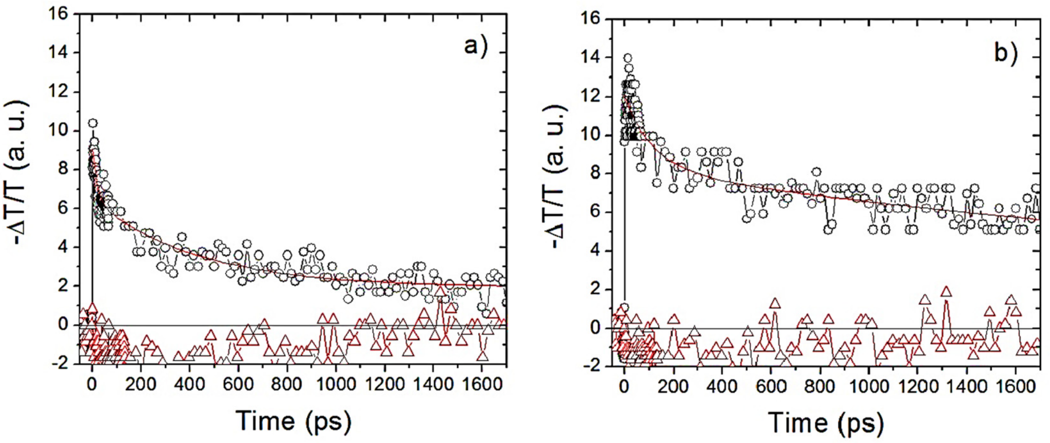

Figure S3. Transient absorption curves for: (**a**) MEH-PPV:$PC_{61}BM$ (1:2 by weight); (**b**) MEH-PPV:$PC_{61}BM$:M1 (1:2:1 by weight). Pump wavelength of 400 nm and energy of 0.7 μJ/pulse; probe wavelengths of 650 nm (circles) and 550 nm (triangles); solid red lines are fits to a vertically offset biexponential function; black line at $-\Delta T/T = 0$ is a guide to the eye.

Table S1. Time decay constants for TA signal for the different studied samples.

Probe Wavelength (nm)	Time Constants of biexpontial decay			
	MEH-PPV:$PC_{61}BM$		MEH-PPV:$PC_{61}BM$:M1	
	Short τ_1 (ps)	Long τ_2 (ns)	Short τ_1 (ps)	Long τ_2 (ns)
650	20	0.43	82	1.53
735	55	0.51	74	1.64
900	13	1	NA	2.32

When the probe beam is tuned to probe the MEH-PPV polaronic band (Figure S3b), we observe similar trends as with the case of P1. When M1 is present, we observe an increase in the time constants with respect to the sample without it. At 900 nm we could not measure the short constant (τ_1) for the sample with M1; nevertheless, the results in Table S1 for the sample without M1 indicate that the excitons at the interface are dissociated relatively fast ($\tau_1 = 13$ ps).

Table S2. Electrochemical HOMO and LUMO levels and GAP of MEH-PPV, M1 and $PC_{61}BM$.

Electrochemical	MEH-PPV	M1	$PC_{61}BM$
LUMO (eV)	−2.85	−3.66	−3.7
HUMO (eV)	−5.15	−5.22	−6.1
GAP (eV)	2.3	1.56	2.4
